# Early intervention for schizophrenia: a pathway to improved clinical outcomes

**DOI:** 10.1017/S1092852925100394

**Published:** 2025-07-10

**Authors:** Takesha Cooper

**Affiliations:** Department of Psychiatry and Behavioral Sciences, University of Nevada, Reno School of Medicine, Renown Health, Reno, NV, USA

**Keywords:** Early Intervention, schizophrenia, outcomes, youth, psychosis

## Abstract

Roger was a 60-year-old man living with both HIV and schizophrenia who was admitted to the hospital for treatment of a chronic obstructive pulmonary disease exacerbation. He was referred to the psychiatry consultation-liaison team due to persistent psychotic symptoms that had not responded to multiple antipsychotic trials. Roger’s psychiatric history revealed a diagnosis of schizophrenia in early adulthood, marked by hallucinations and delusions of grandeur. Over the next 4 decades, he cycled through jails, prisons, shelters, and periods of homelessness. Though intermittently connected with outpatient care, his illness remained poorly controlled.

## Clinical Vignette: The Life and Loss of “Roger”

Roger was a 60-year-old man living with both HIV and schizophrenia who was admitted to the hospital for treatment of a chronic obstructive pulmonary disease exacerbation. He was referred to the psychiatry consultation-liaison team due to persistent psychotic symptoms that had not responded to multiple antipsychotic trials. Roger’s psychiatric history revealed a diagnosis of schizophrenia in early adulthood, marked by hallucinations and delusions of grandeur. Over the next 4 decades, he cycled through jails, prisons, shelters, and periods of homelessness. Though intermittently connected with outpatient care, his illness remained poorly controlled.

At age 55, after being deemed not competent to stand trial following an assault, Roger was sent to the state hospital. During that stay, he assaulted a nurse while experiencing active psychosis and was subsequently transferred to jail, where he served a short sentence. After his release, he returned to homelessness, and was later found emaciated and struggling to breathe on the street. Findings upon hospital admission included: oxygen saturation at 88% on room air, HIV viral load of 100 000 copies/mL (previously undetectable when treated), and significant leukopenia.

While Roger’s pulmonary and infectious disease conditions improved over a month-long hospitalization, his psychosis remained unremitting despite treatment with several antipsychotics, including both oral and long-acting injectable formulations of typical and atypical agents (eg, haloperidol, risperidone, quetiapine, prolixin). He continued to exhibit complex delusions, including the belief that Jesus had impregnated him and infected him with HIV. Despite this, Roger was deeply engaged and responsive to compassionate care. The treatment team advocated for his continued hospitalization, even after his medical issues had stabilized, due to the lack of a psychiatric inpatient unit willing to manage his HIV and history of aggression.

Although clozapine had never been trialed—likely due to concerns about neutropenia in the context of HIV—this option was eventually pursued following repeated denials from skilled nursing facilities and complex discharge planning challenges. Within 3 weeks of clozapine initiation and titration to 350 mg daily, Roger showed marked improvements in aggression, psychosis, and adherence to his HIV medications. Placement was finally secured at a facility out of state for individuals with complex psychiatric and medical comorbidities. Tragically, Roger died in the hospital before transfer could occur. An autopsy is pending.

The psychiatric team expressed gratitude that Roger did not die alone on the street—but mourned the missed opportunities throughout his life. His story underscores the urgent need for systemic change.

## Introduction

Schizophrenia is a chronic and severe mental disorder that affects approximately 1% of the global population. Characterized by profound disruptions in thinking, perception, and behavior, it often leads to significant impairments in social and occupational functioning. Traditionally, schizophrenia has been associated with a deteriorating course; however, contemporary research underscores the potential for improved outcomes through early intervention strategies. This article explores the rationale, components, and benefits of early intervention, emphasizing its critical role in enhancing prognosis and recovery.^1^

## Epidemiology, onset, and early intervention

Schizophrenia typically manifests in late adolescence to early adulthood, with a median age of onset in the early to mid-20s for males and late 20s for females.^2^ The period preceding the first psychotic episode—the prodromal phase—is marked by subtle changes in cognition, mood, and behavior. Individuals may experience social withdrawal, unusual thoughts, and a decline in daily functioning. The duration of untreated psychosis (DUP)—the time from symptom onset to initiation of treatment—is often prolonged. A longer DUP has been consistently associated with poorer clinical and functional outcomes.^3^ Early intervention can fundamentally alter the trajectory of schizophrenia and are most effective when they are multidisciplinary, personalized, and sustained over time. Reducing DUP improves symptomatic and functional outcomesf, fewer relapses, and higher quality of life.^4^ There is also neurobiological evidence suggesting that early intervention may preserve brain structure and function by leveraging neuroplasticity during a critical developmental window.^5^ ^,^^6^ As a result, early-phase treatment may be more effective and better tolerated. The most effective early intervention programs are designed to address the full spectrum of needs that arise in early psychosis, including symptom management, functional recovery, family engagement, and social reintegration.

## Models of care and program examples

Several international and US-based care models provide frameworks for early intervention services, demonstrating replicable success across diverse healthcare systems. At their core, early intervention programs for schizophrenia typically offer a comprehensive, multidisciplinary approach designed to address both clinical and functional recovery. Core components include low-dose antipsychotic medication, individual and group psychotherapy (often using cognitive behavioral therapy), family education and support, case management, and assistance with school, work, and social reintegration when ready. These services are developmentally tailored and delivered in a coordinated manner, often in community or outpatient settings, with a strong emphasis on engaging both the patient and their family early in the course of illness.

**Coordinated specialty care (CSC)** is the leading model in the United States and was validated through the National Institute of Mental Health (NIMH)-funded Recovery After an Initial Schizophrenia Episode (RAISE) study. CSC teams include psychiatrists, therapists, employment and education specialists, case managers, and peer support specialists. This model emphasizes shared decision-making, individualized care plans, and community integration.^7^ Services are usually delivered in outpatient settings and tailored to developmental stages, recognizing the unique challenges of psychosis in adolescence and early adulthood.

**The Early Psychosis Prevention and Intervention Centre (EPPIC)** in Melbourne, Australia, represents one of the earliest and most influential international models. EPPIC offers time limited but intensive services for youth aged 15–24, integrating medical, psychological, vocational, and social interventions. Its success has catalyzed similar programs across Europe and Asia, reinforcing the scalability and global relevance of early psychosis care.^8^

**Assertive community treatment (ACT)**, while not specific to early intervention, is often integrated into early psychosis services for individuals with high acuity or co-occurring conditions. ACT provides multidisciplinary, community-based care with 24/7 availability, minimizing the need for hospitalization and addressing both clinical and social determinants of health.^9^ When combined with CSC principles, ACT can be particularly effective for individuals with housing instability, trauma histories, or frequent psychiatric hospitalizations. These three models share a commitment to early, assertive, and sustained intervention that prioritizes recovery and community reintegration. The key to success lies in accessibility, coordination, and person-centered care.

## Medication and therapy

Medication remains a foundational element, with low-dose, second-generation antipsychotics typically recommended for first-episode psychosis to minimize side effects and maximize adherence.^10^ Medication choice is guided by clinical presentation, patient preference, side effect profile, and family history. The goal is not merely symptom suppression but optimizing tolerability to promote sustained engagement. Clozapine is also approved to reduce the risk of recurrent suicidal behavior in patients with schizophrenia or schizoaffective disorder who are considered at chronic risk for reexperiencing suicidal behavior.^11^ On February 24, 2025, the US Food and Drug Administration officially ended the mandatory requirements associated with the Clozapine Risk Evaluation and Mitigation Strategy (REMS) program. This followed a recommendation from the Psychopharmacologic Drugs Advisory Committee, which concluded that the REMS no longer contributed significantly to the safe use of clozapine and was an unnecessary barrier to access.^12^ In addition to medication, psychotherapeutic interventions are critical. Cognitive behavioral therapy (CBT) has demonstrated efficacy in reducing positive symptoms, enhancing coping strategies, and delaying or preventing relapse.^13^ Family psychoeducation equips caregivers with the tools to support recovery while reducing expressed emotion—an identified predictor of relapse.^14^

## Barriers to early intervention

Despite the demonstrated effectiveness of early intervention programs, multiple barriers hinder their widespread implementation. These barriers span structural, clinical, cultural, and policy domains, contributing to delays in diagnosis and treatment.

**Stigma** continues to be one of the most significant barriers to accessing mental health care. Many individuals and families hesitate to seek support due to fears of being labeled, facing discrimination, or experiencing internalized shame. This challenge is especially pronounced among youth and young adults, who may misinterpret early symptoms or intentionally hide them from caregivers and health professionals.^15^ Misconceptions persist, including the belief, that mental illness is not as legitimate as physical illness and that individuals should simply “try harder” to overcome conditions like depression. To address these harmful attitudes, coordinated public and provider education efforts are essential to normalize help-seeking behaviors and reduce stigma-related delays in care.

**Misdiagnosis and underrecognition** frequently occur, especially in primary care and educational settings. Early signs of psychosis—such as social withdrawal, anxiety, or mild paranoia—can be misattributed to typical adolescent behavior, mood disorders, or substance use. As a result, many individuals remain undiagnosed until symptoms become severe, missing the opportunity for preventive intervention.^16^

**Access limitations,** including geographic disparities and insurance coverage gaps, further restrict timely intervention. Rural areas often lack specialized early psychosis programs, forcing families to travel long distances or settle for fragmented care. Financial barriers, particularly in underinsured or uninsured populations, compound these challenges.

**Cultural and systemic inequities** exacerbate disparities in access and quality of care. Black, Indigenous, and People of Color are disproportionately subject to coercive pathways to care, including involuntary hospitalization and involvement with law enforcement, rather than voluntary, recovery-oriented services.^17^ Language barriers, lack of accountability for past harms by medical institutions, and lack of culturally informed providers further alienate marginalized communities.

Addressing these barriers requires a multifaceted approach that includes workforce development, policy advocacy, and the expansion of culturally responsive care. Without deliberate strategies to close these gaps, early intervention will remain inaccessible to those who might benefit most.

## Reflections on missed opportunities

Roger’s life could have unfolded very differently had our healthcare, social, and legal systems been better equipped to respond to severe mental illness with urgency, compassion, and evidence-based care. If he had received early intervention at the time of his first psychotic break—rather than entering a decades-long cycle of incarceration, homelessness, and episodic treatment—he may have enjoyed stability, independence, and connection. Instead of being criminalized for behavior rooted in psychosis, he could have received coordinated, community-based care that addressed both his mental health and social needs.

Importantly, Roger never received a trial of clozapine, the gold standard for treatment-resistant schizophrenia, until the final months of his life—even after multiple medication failures over several decades. This delay was likely due to systemic inertia and concerns about side effects in the context of comorbid HIV, despite evidence that clozapine can be safely administered in such cases with proper monitoring. His case exemplifies the consequences of failing to follow evidence-based treatment algorithms and illustrates the profound costs of fragmented care.

In a more humane and responsive system—one that values early identification, comprehensive intervention, and respect for the dignity of those living with schizophrenia—Roger’s outcome could have been drastically different. His story challenges us to confront the moral and structural failures that allow individuals with treatable illnesses to fall through the cracks. It is a sobering reminder that early intervention is not merely a clinical strategy—it is a call to action rooted in equity, ethics, and compassion.

## References

[1] McGorry PD, Killackey E, Yung AR. Early intervention in psychosis: concepts, evidence and future directions. World Psychiatry. 2008;7(3):148–156. 18836582 10.1002/j.2051-5545.2008.tb00182.x PMC2559918

[2] American Psychiatric Association. Diagnostic and Statistical Manual of Mental Disorders. 5th ed. American Psychiatric Publishing; 2013.

[3] Marshall M, Lewis S, Lockwood A, et al. Association between duration of untreated psychosis and outcome in cohorts of first-episode patients: a systematic review. Arch Gen Psychiatry. 2005;62(9):975–983. doi:10.1001/archpsyc.62.9.975. 16143729

[4] Penttilä M, Jääskeläinen E, Hirvonen N, Isohanni M, Miettunen J. Duration of untreated psychosis as a predictor of long-term outcome in schizophrenia: systematic review and meta-analysis. Br J Psychiatry. 2014;205(2):88–94. doi:10.1192/bjp.bp.113.127753. 25252316

[5] Birchwood M, Todd P, Jackson C. Early intervention in psychosis: the critical period hypothesis. Br J Psychiatry. 1998;172(33):53–59. doi:10.1192/S000712500029768. 9764127

[6] Cooper T, Seigler MD, Stahl S. Rapid onset brain plasticity at novel pharmacologic targets hypothetically drives innovations for rapid onset antidepressant actions. J Psychopharmacol. 2023;37(3):242–247. doi:10.1177/02698811231161243. 36988262 PMC10076337

[7] Kane JM, Robinson DG, Schooler NR, et al. Comprehensive versus usual community care for first-episode psychosis: 2-year outcomes from the NIMH RAISE Early Treatment Program. Am J Psychiatry. 2016;173(4):362–372. doi:10.1176/appi.ajp.2015.15050632. 26481174 PMC4981493

[8] McGorry PD, Yung AR, Phillips LJ. The “close-in” or ultra high-risk model: a safe and effective strategy for research and clinical intervention in prepsychotic mental disorder. Schizophr Bull. 2002;28(3):485–496. doi:10.1093/oxfordjournals.schbul.a006989. PMC3677160 14989414

[9] Dieterich M, Irving CB, Park B, Marshall M. Intensive case management for severe mental illness. Cochrane Database Syst Rev. 2017;1:CD007906. doi:10.1002/14651858.CD007906.pub3. 20927766 PMC4233116

[10] Kahn RS, Fleischhacker WW, Boter H, et al. Effectiveness of antipsychotic drugs in first-episode schizophrenia and schizophreniform disorder: an open randomized clinical trial. Lancet. 2008;371(9618):1085–1097. doi:10.1016/S0140-6736(08)60486-9. 18374841

[11] US Food and Drug Administration. Clozaril [prescribing information]. 2010. https://www.accessdata.fda.gov/drugsatfda_docs/label/2010/019758s062lbl.pdf.

[12] US Food and Drug Administration. Frequently asked questions about the Clozapine REMS modification; 2024. https://www.fda.gov/drugs/postmarket-drug-safety-information-patients-andproviders/frequently-asked-questions-clozapine-rems-modification. Accessed April 30, 2025.

[13] Bighelli I, Rodolico A, García-Mieres H, et al. Psychosocial and psychological interventions for relapse prevention in schizophrenia: a systematic review and network meta-analysis. Lancet Psychiatry. 2021;8(11):969–980. doi:10.1016/S2215-0366(21)00243-1. 34653393

[14] Dixon L, McFarlane WR, Lefley H, et al. Evidence-based practices for services to families of people with psychiatric disabilities. Psychiatr Serv. 2001;52(7):903–910. doi:10.1176/appi.ps.52.7.903. 11433107

[15] Corrigan PW. How stigma interferes with mental health care. Am Psychol. 2004;59(7):614–625. doi:10.1037/0003-066X.59.7.614. 15491256

[16] Singh SP, Grange T. Measuring pathways to care in first-episode psychosis: a systematic review. Schizophr Res. 2006;81(1):75–82. doi:10.1016/j.schres.2005.09.018. 16309892

[17] Oluwoye O, Kriegel LS, Alcover KC, McDonell MG. Racial and ethnic disparities in first-episode psychosis: a review of the literature. Psychiatr Serv. 2021;72(5):567–577. doi:10.1176/appi.ps.202000353.

